# LMP1-EBV Gene Deletion Mutations and HLA Genotypes of Nasopharyngeal Cancer Patients in Vietnam

**DOI:** 10.3390/pathophysiology30010001

**Published:** 2023-01-11

**Authors:** Cua Thi Hong Trinh, Dung Ngoc Tran, Linh Thi Thao Nguyen, Nghia Tin Tran, Minh Trinh Gia Nguyen, Vy Tran Phuong Nguyen, Nhung Thi Hong Vu, Khanh Duy Dang, Kha Van Vo, Hoa Chieu Chau, Phi Thi Phi Phan, Mai Huynh Truc Phuong

**Affiliations:** 1 Department of Pathophysiology and Immunology, Can Tho University of Medicine and Pharmacy, Can Tho 900000, Vietnam; 2 Department of Pharmacology and Clinical Pharmacy, Can Tho University of Medicine and Pharmacy, Can Tho 900000, Vietnam; 3 Can Tho Oncology Hospital, Can Tho 900000, Vietnam; 4 Can Tho Ear Nose Throat Hospital, Can Tho 900000, Vietnam; 5 Department of Physiopathology & Immunology, Ha Noi Medical University, Ha Noi 100000, Vietnam; 6 Faculty of Medicine, Can Tho University of Medicine and Pharmacy, Can Tho 900000, Vietnam

**Keywords:** nasopharyngeal carcinoma, LMP1-EBV gene, HLA genotype

## Abstract

Nasopharyngeal carcinoma (NPC) is the most common cancer among head and neck cancers in Vietnam. We aimed to identify the rate of a 30 bp deletion mutation of the LMP1-EBV gene in nasopharyngeal biopsy tissue samples, the HLA genotypes of NPC patients, and the relationship between these two targets. Patients with NPC at Can Tho Oncology Hospital from September 2014 to December 2018 were selected. A length of 30 bp of the del-LMP1-EBV gene was analyzed using a PCR technique, and the HLA genotypes in patients’ blood samples were analyzed with PCR-SSO technology. HLA-B*15 gene carriers had the highest risk of 30 bp LMP1-EBV gene deletion mutation, which was found in 51 out of 70 patients (72.9%). Carriers of the HLA-B*15 allele had a 4.6-fold increased risk of a 30 bp del-LMP1-EBV gene compared with non-carriers of this allele. The initial identification of NPC was related to the 30 bp del-LMP1-EBV gene and high frequencies of the -A*02, -B*15, -DRB1*12, -DQB1*03, and -DQA1*01 HLA alleles. Our study results suggest an association of the 30 bp del-LMP1-EBV gene and the HLA-B*15 allele with NPC susceptibility.

## 1. Introduction

Malignant tumors called nasopharyngeal carcinomas mostly develop from the layer of differentiated epithelial cells that covers the nasopharynx. They are one of Vietnam’s ten most common malignancies among head and neck cancers [1,2]. The human leukocyte antigen (HLA) genetic component of a patient’s vulnerability to the disease, Epstein–Barr virus (EBV) infection, and the effect of dietary components are the three categories of pathogenic variables that are associated with the pathophysiological features of NPC. The latent membrane protein 1 gene of EBV (LMP1-EBV) is one of the EBV genes discovered in most nasopharyngeal biopsies of patients with NPC [1,3,4,5,6]. It is a gene encoding the membrane protein LMP1, a unique EBV gene product capable of causing transformation, inhibiting epithelial cell differentiation, and acting as an initiating protein for the malignant transformation of cells [4,6,7,8]. In addition, the evasion of the EBV immune response by mutations in the LMP1-EBV gene, especially the 30 bp deletion mutation of the LMP1-EBV gene, produces non-immunogenic LMP1 products; it is also the way that EBV initiates tumorigenesis and growth [9,10]. Many studies have also mentioned a second factor related to the biological pathogenesis of NPC: the genetic factor of the HLA gene in susceptible patients with NPC. This factor explains the prevalence of NPC in certain geographical areas [11,12,13]. However, the HLA molecule—a product of the HLA gene—has many functions, including the antigen presentation process of recognizing a host’s immune cells. Therefore, another problem is the relationship between HLA gene sensitivity and the mutation status of the LMP1-EBV gene, especially the 30 bp gene deletion mutation of the LMP1-EBV gene, which is a common mutation in nasopharyngeal cancer that previous studies have recognized.

Our study’s objectives are to ascertain the frequency of HLA genotypes in nasopharyngeal cancer patients treated at a Vietnam oncology hospital, the rate of 30 bp gene deletion mutations in the LMP1-EBV gene in nasopharyngeal biopsy samples, and the relationship between these two variables.

## 2. Materials and Methods

### 2.1. Materials

Nasopharyngeal biopsy tissue samples of NPC patients were obtained from Can Tho Oncology Hospital in the Mekong Delta region (from September 2014 to December 2018), as well as blood samples from NPC patients.

#### 2.1.1. Selection Criteria

Patients with NPC who were treated at Can Tho Oncology Hospital were included, with no age or gender restrictions. For the nasopharyngeal biopsy tissue samples, fresh, unprocessed biopsy tissue was collected in an amount of 0.5–4 mg. Nasopharyngeal biopsy tissue samples embedded in paraffin blocks were used in cases when fresh biopsy tissue samples did not reach the standard amount. Collected tissue samples were embedded in paraffin blocks. The histopathological results for the same patient were embedded in a paraffin block for the duration of tissue sample collection, and reading of the results did not exceed 4 weeks after collection (about 10 slices, with a thickness of 5 µm). Blood samples of NPC patients showed the presence of the LMP1-EBV gene in the nasopharyngeal biopsy tissue sample.

#### 2.1.2. Exclusion Criteria

The exclusion criteria included patients with recurring nasopharyngeal cancer, cancer revisiting periodically during treatment, those residing in the study region for less than one year, those not living in the provinces of the Mekong Delta, or those who did not agree to participate in the study.

### 2.2. Methods

#### 2.2.1. Study Design

**Figure 1:**
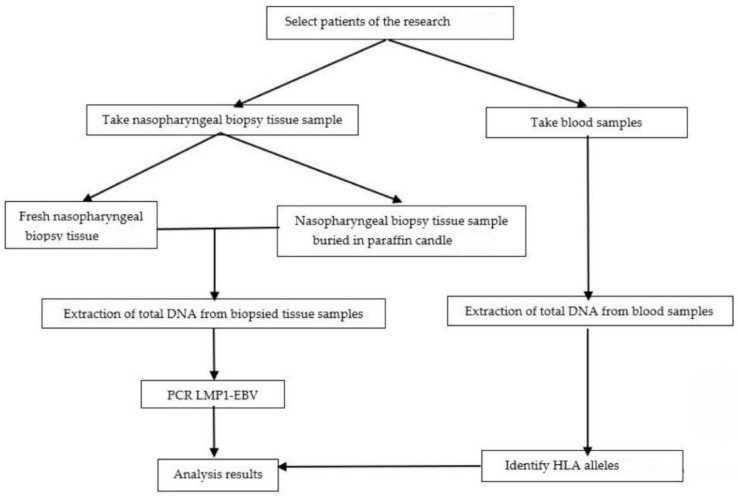
Study chart.

Figure 1 depicts a cross-sectional description of the study and analysis.

#### 2.2.2. Sample Selection

We selected 108 samples and collected all the samples that matched the sample selection criteria during the study period (objective 1), as well as randomly selecting a single sample (objective 2).

### 2.3. Study Equipment

#### 2.3.1. DNA extraction chemicals

For chemical DNA extraction from the biopsy tissue, an Invisorb^®^ Spin Tissue Mini Kit (Stratec company, Birkenfeld city, Germany) was used.

For chemical DNA extraction from the whole blood samples, an Invisorb^®^ Spin Blood Mini Kit (Stratec company, Birkenfeld city, Germany) was used.

#### 2.3.2. PCR Reaction Chemicals

The following were used for DNA amplification: Taq DNA polymerase 5U (Promega); an LMP1-EBV gene primer pair with a sequence from 168373–168174 (Phu Sa biochemical company); buffer (Taq buffer with KCl and MgCl_2_) (Promega); dNTP (dATP, dCTP, dGTP, and dTTP) (Promega); and sterile, distilled water.

The chemical electrophoresis amplification products included the following: agarose (Bioline company); 1 × TBE electrophoresis buffer (Tris base: 10.8 g, boric acid: 5.5 g/L, and 0.5M EDTA: 4 mL); loading buffer (3 mL glycerol (30%), 20 mg bromophenol blue (0.25%), 200 mM Tris, 20 mM Na2EDTA, and 10 mL distilled water stored at 4 °C); 10 mg/mL safe view (Bioline); and 100 bp DNA Ladder (Fermentas company).

#### 2.3.3. Chemicals Required for Automated Gene Sequencing

The following kits were used for automated gene sequencing: a BigDye^®^ Terminator v3.1 Cycle Sequencing Kit (Applied Biosystems) and a DNA purification GenJet Gel Extraction Kit (Thermo Scientific) using agarose gel.

#### 2.3.4. Chemicals Needed for PCR-SSO Technique to Determine HLA Gene Type Using One Lambda

The following were used in the PCR-SSO technique to determine the HLA gene types of patients:

LABType CWD Class IA locus typing;

LABType CWD Class IB locus typing;

LABType CWD Class II DRB1 typing;

LABType SSO Class II DQB1/DQA1 typing;

LT-SAPE (PE-conjugated streptavidin);

Taq DNA polymerase.

### 2.4. Study Content and Techniques

#### 2.4.1. Techniques

The rate of 30 bp deletion mutation of the LMP1-EBV gene in nasopharyngeal biopsy samples of patients with NPC was assessed by performing a PCR technique at the molecular biology laboratory of Can Tho University of Medicine and Pharmacy. The process included the following steps:

(1) Extracting the total DNA from biopsy tissue samples (including viral genomes) using an Invisorb^®^ Spin Tissue Mini Kit, measuring DNA concentration (OD_260_), and evaluating the purity of the DNA by calculating OD_260_/OD_280_ (1.6–2.1) with a BioDrop system.
(2) Performing a PCR reaction with an LMP1-EBV primer pair with sequences at the following position: 168,373 (5′-CTA GCG ACT CTG CTG GAA AT-3′) and 168,174 (5′-CGC GGA TCC TTA GTC ATA GTA GCT TAG-3′). The composition used for the PCR reaction was 2.5 µL DNA, 3 µL dNTPs, 1 µL each primer, 3 µL MgCl_2_, 0.5 µL Taq DNA polymerase, 5 µL buffer, and 34 µL distilled water. The total volume was 50 µL. The heat cycle was as follows: 95 °C for 7 min, 35 cycles of 94 °C for 1 min and 30 s, 55 °C for 1 min, 72 °C for 1 min and 30 s, and 72 °C for 7 min.
(3) Reading the results after 1 h: electrophoresis was performed with 9 µL PCR product on 2% agarose gel in 1 × TBE buffer at a voltage of 50 V.

The research samples’ LMP1-EBV PCR products were positive, indicating the presence of the LMP1-EBV gene when an electrophoresis line product with a size of 230 bp or 200 bp was present on the gel. For a sample size of 200 bp, samples could have a 30 bp deletion mutation of the LMP1-EBV gene. As a result, we determined the frequency of the 30 bp deletion mutation of the LMP1-EBV gene by dividing the total number of samples with 200 bp of amplification products by the number of complete models with the LMP1-EBV gene present (230 bp or 200 bp). In addition, the study verified the 30 bp deletion mutation from the 200 bp amplification products using an LMP1-EBV gene-sequencing technique with an ABI PRISM 3730xl Genetic Analyzer automated sequencer system.

#### 2.4.2. Study Content

To determine the prevalence of the HLA gene in blood samples of patients with NPC, we performed a PCR-SSO technique using a LABScan3D™ system (Luminex FLEXMAP 3D) with LABType™XR and CWD Typing Test chemicals. We analyzed the results using HLA Fusion™ LABType software^®^ at the immuno-assay unit of Cho Ray Hospital to identify HLA alleles from the blood samples of the study patients. The frequency of an HLA gene allele was recorded as the occurrence of a particular HLA allele at a high rate compared with the occurrence of other HLA alleles in the total HLA alleles of the survey of the HLA gene locus.

### 2.5. Statistical Analysis

The collected study data were entered into the EpiData 3.1 program, and statistical processing was performed using Stata 10.0 software. We used a descriptive statistics algorithm to describe the ratio values of the variables and tested the hypothesis with a chi-squared test.

### 2.6. Research Ethics

The study was approved by the Proposal Defense Council of the Institute of Biotechnology, Research, and Development at Can Tho University.

The study was conducted only after receiving permission from the Scientific Research Council and the Board of Directors of Can Tho Oncology Hospital.

The steps to conduct the study were approved by the dean of the department, the treating doctor at the department, and the patients’ voluntary consent to participate in the study (obtained through a consent form to participate in the survey). The examination procedure was approved when a patient’s condition was stable.

During the implementation of the study, the patients’ rights were guaranteed: encryption for patient information was confidential, patients could refuse to participate at any time during the research process, and study participation did not increase any costs of patient treatment.

## 3. Results

During the implementation of the study, we recorded 108 NPC cases treated at Can Tho Oncology Hospital that met the sample selection criteria. From 108 participants, a total of 70 nasopharyngeal biopsy samples with the presence of the LMP1-EBV gene were found in the electrophoresis analysis (including 38 fresh biopsy tissue samples and 70 biopsy tissue samples embedded in paraffin blocks). The results for the nasopharyngeal objectives are presented as follows.

### 3.1. Prevalence of LMP1-EBV Gene Mutations in Nasopharyngeal Biopsy Tissue Samples of Patients with NPC

**Figure 2:**
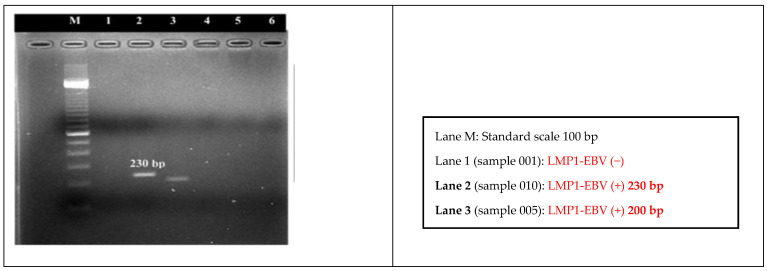
Electrophoresis line image of LMP1-EBV gene amplification product with a size of 200 bp (Lane 3, sample 005) on 2% agarose gel.

Among 70 nasopharyngeal biopsy tissue samples from patients with the presence of the LMP1-EBV gene found by electrophoresis, the study detected 72.9% (51/70) of the amplification products with 200 bp (expressing a 30 bp deletion mutation of the LMP1-EBV gene) (Table 1 and Figure 2).

**Figure 3:**
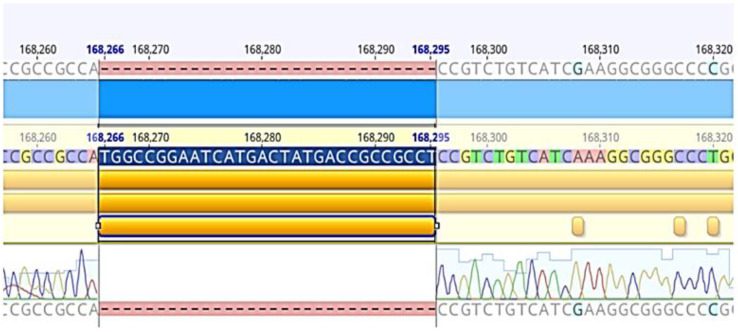
Results for 30 bp deletion mutation of LMP1-EBV gene in sample 111 (compared with B95-8 standard strain (Genbank V01555) at position 168,266–168,295).

Among 33 nasopharyngeal biopsy tissue samples with the presence of the LMP1-EBV gene that underwent gene sequencing, 25/33 samples were detected to have a 30 bp gene deletion mutation (168,266–168,295), with a percentage of 75.8% (Table 2 and Figure 3).

### 3.2. Prevalence of HLA Gene in Blood Samples of Patients with NPC

When selecting samples for HLA gene determination in nasopharyngeal cancer patients, we randomized only 30 patients with nasopharyngeal cancer who met the nasopharyngeal cancer patient criteria, and we identified the presence of the LMP1-EBV gene in nasopharyngeal biopsy tissue samples to assess objective 1.

The study detected 7 types of HLA-A alleles and 16 types of HLA-B alleles for class-I HLA genes. The HLA-A allele with the highest frequency was -A*02 (40.4%), followed by -A*11 (21.2%), -A*24 (21.2%), and -A*33 (9.6%). Similarly, the HLA-B allele with the highest frequency was -B*15 (25.0%), followed by -B*46 (23.1%), -B*38 (9.6%), and -B*07 (7.7%) (Table 3).

The study detected 12 types of HLA-DRB1 alleles, 5 types of HLA-DQB1 alleles, and 5 types of HLA-DQA1 alleles. The class-II HLA alleles that appeared frequently in the studied patients were HLA-DRB1*12 (17.3%) and -DRB1*09 (13.8%); -DQB1*03 (44.7%), -DQB1*05 (21.4%), and -DQB1*06 (17.9%); and HLA-DQA1*01 (35.7%), -DQA1*03 (28.6%), and -DQA1*06 (21.4%) (Table 4).

### 3.3. Association between 30 bp Deletion Mutation Rate of LMP1-EBV Gene and Frequency of HLA Alleles

There was a statistically significant difference in the HLA-B*15 alleles of the groups with and without 30 bp deletion mutations of the LMP1-EBV gene, with OR = 4.64 and *p* < 0.05. This meant that patients carrying the HLA-B*15 allele had an LMP1-EBV 30 bp deletion mutation rate about 4.6 times higher than those who did not carry this allele. For the remaining alleles of -A*02, -A*11, -A*24, -B*38, -B*46, -DRB1*12, -DRB1*09, -DQB1*03, and -DQA1*01, there were no statistical differences between the two groups (Table 5).

## 4. Discussion

By using the PCR technique with an LMP1 primer pair (168,373–168,174), we detected 70/108 (64.81%) nasopharyngeal biopsy tissue samples of the patients with the presence of the LMP1-EBV gene (230 bp and 200 bp). Amplification products of 200 bp in size were found in 72.9% (51/70) of the 70 samples from the LMP1-EBV gene patients (Figure 1). As a result, the PCR method detected 72.9% of the 30 bp deletion mutations of the LMP1-EBV gene.

As we continued to compare our results with the studies of other authors worldwide, we saw differences in the rates of 30 bp deletion of the LMP1-EBV gene among studies in different geographical regions. For example, in China, the rate of 30 bp deletion mutations of the LMP1-EBV gene was 84% (36/43) (Zhang et al., 2002) [14]; Malaysia also had a similar rate of 84% (21/25) (Tan et al., 2003) [15]; Morocco (north Africa) had a rate of 84% (51/61) 30 bp deletion mutations of the LMP1-EBV gene (Dardari et al., 2006) [16]; and 6.67% (28/42) was the proportion found in a study in Tunisia (northeast Africa) (Boutheina et al., 2006) [17]. In Asia, the rate of 30 bp deletion mutations of the LMP1-EBV gene in NPC patients accounted for the highest rate (79%), followed by that in the Americas (64%) and Europe, with north Africa having the lowest rate (59%).

Using sequencing methods and the analysis results of 25 LMP1-EBV gene sequences compared with the EBV B95-8 reference strain (Genbank V01555), we randomly selected 25 samples with sizes of 200 bp and compared them with the clear electrophoresis lines in 51 samples showing the presence of the LMP1-EBV gene. There were 30 bp deletion mutations at the 168,266–168,295 position (Figure 2).

Therefore, when both PCR electrophoresis and LMP1-EBV gene sequencing were performed, the results corresponded completely, with 30 bp deletion mutations of the LMP1-EBV gene in patients with NPC.

We compared the results obtained in this study with previous results from Tran Ngoc Dung (2000) [18], who studied class-I and -II HLA alleles in 47 nasopharyngeal cancer patients from northern Vietnam using cytotoxicity based on Terasaki’s technique and indirect immunofluorescence and showed that there were 11 types of HLA-A alleles with high frequencies, including -A*02 (30%), -A*11 (20%), -A*25 (17%), and -A*24 (9.4%) [18]. Compared with the results of this study, there are similarities in the frequencies of the -A*02, -A*11, and -A*24 alleles, which also accounted for the alleles with the highest proportions in the studied patients. However, there are differences in NPC patients from two different geographical regions of Vietnam. The assessment of allele frequency for locus A in Vietnamese NPC patients resulted in the -A*02, -A*11, and -A*24 alleles showing high frequency and these are the HLA alleles that often appear in individuals of Asian descent.

In a study of 203 NPC patients in Taiwan, researchers found the highest frequencies of HLA-A alleles were for -A*02 (37.45%), -A*11 (29.8%), -A*24 (16.5%), and -A*33 (11.1%) (Hildesheim et al., 2002) [19]. In southern China, alleles with the highest frequencies were -A*02 (38.62%), -A*11 (23.74%), and -A*33 (20.51%) (Tang et al., 2010) [20]. A study in northern China on the association between HLA alleles in 132 nasopharyngeal cancer patients found 11 types of HLA-A alleles; those with the highest frequencies were -A*02 (62.1%), -A*24 (42.4%), -A*11 (27.3%), and -A*33 (16.7%) (Wang and Wang, 2014) [12]. In another study in Tunisia (north Africa), for 28 patients with NPC, 19 HLA-A alleles were found, and the highest frequencies of HLA-A alleles were for -A*02 (39.28%), -A*24 (25%), -A*30 (25%), and -A*26 (21.42%) (Nehla et al., 2017) [21].

In the HLA-B locus, our study detected 16 alleles (Results Table). We compared this with the results of Tran Ngoc Dung (2000) [18] from a study conducted in northern Vietnam in which there were 20 HLA-B alleles detected, and those with high frequencies were HLA-B*17 (21%), -B*62 (13%), -B*49 (9.4%), and -B*57 (8.3%) This was similar to our study results for eight HLA-B alleles: -B*15, -B*07, -B*18, -B*40, -B*44, -B*51, -B*57, and -B*58. However, two alleles (-B*46 and -B*38) accounted for a relatively high proportion in our study but were not found in the study of Tran Ngoc Dung (2000) [18]. In contrast, our study did not detect HLA-B*17, -B*62, and -B*49. In healthy individuals, HLA-B alleles are more diverse than HLA-A alleles; one study found 412 types of HLA-B alleles (Nguyen Dinh Binh, 2014) [22]. Because of this diversity, there are differences in the frequencies of HLA-B alleles between domestic and foreign studies.

Some foreign studies on HLA-B alleles in patients with NPC have also given similar results. Specifically, the HLA-B allele results in 203 cancer patients in Taiwan showed that the highest frequencies of HLA-B alleles were for HLA-B*46 (18.0%), -B*58 (12%), -B*15 (11.2%), and -B*38 (4.6%) (Hildesheim et al., 2002) [19]. A study on the association between HLA alleles in 132 NPC patients in northern China discovered 25 types of HLA-B alleles, and the alleles with high frequencies were HLA-B*40 (31.1%), -B*46 (30.3%), -B*15 (19.7%), -B*13 (15.2%), -B*58 (13.6%), and -B*07 (4.5%) (Wang and Wang, 2014) [12]. In another study in Tunisia (north Africa) on 25 NPC patients, there were 23 identified HLA-B alleles, and those with the highest frequencies were HLA-B*50 (32%), -B*51 (32%), -B*44 (20%), -B*08 (16%), -B*27 (16%), and -B*52 (16%) (Nehla et al., 2017) [21].

In short, the results for the HLA-B alleles in this study are similar to those of the above studies for some alleles, such as -B*46, -B*15, and -B*38. However, the frequency of encountering each HLA-B allele varied, which indicated the diversity of HLA-B alleles in patients with NPC.

The results of this study recorded 12 types of HLA-DRB1 allele. The high frequency of alleles were for -DRB1*12 (17.3%), -DRB1*09 (13.8%), -DRB1*04 (12.1%), -DRB1*08 (12.1%), -DRB1*15 (12.1%), -DRB1*07 (10.3%), and -DRB1*14 (10.3%). These results are similar to those of another study from Vietnam (Tran Ngoc Dung, 2000) for eight alleles (-DR*03, -DR*04, -DR*07, -DR*08, -DR*09, -DR*10, -DR*12, and -DR*15). However, there are differences for two alleles (-DR*01 (15%) and -DR*11 (12%)) appearing frequently in the other study; our study did not find the -DR*01 and -DR*11 alleles. We continued to compare our results with other studies worldwide. We saw that a study on the association between the HLA alleles of 132 NPC patients in northern China found that HLA-DRB1 alleles with high frequencies of occurrence were -DRB1*09 (35.6%), -DRB1*04 (25.8%), -DRB1*15 (20.5%), -DRB1*07 (22.7%), -DRB1*08 (19,7%), -DRB1*12 (15.9%), and -DRB1*14 (13.6%) (Wang and Wang, 2014) [12]. Moreover, Geng et al. (2016) identified HLA-DRB1 alleles using a PCR-SSP method for 140 patients with nasopharyngeal cancer in China, resulting in an account of the frequencies of HLA-DRB1 alleles in the Uyghur ethnic group of China. The highest ratio was -DRB1*07:01 (25%), followed by -DRB1*03:01, -DRB1*13:01, -DRB1*15:01, and -DRB1*04:01; for the Han ethnic group of China, HLA- DRB1*0901 again accounted for the highest percentage, followed by -DRB1*07:01, -DRB1*15:01, -DRB1*12:01, -DRB1*08:01, and -DRB1*14:01 [23]. There are similarities in the occurrences of HLA-DRB1 alleles (-DRB1*12, -DRB1*09, -DRB1*04, -DRB1*08, -DRB1*15, -DRB1*07, and -DRB1*14) in patients with NPC in our study compared with the results of authors around the world.

For the HLA-DQB1 locus, our study analyzed five types of HLA-DQB1 alleles, with high frequencies of HLA-DQB1 alleles appearing for -DQB1*03 (44.7%), -DQB1*05 (21.4%), -DQB1*06 (17.9%), -DQB1*02 (8.9%), and -DQB1*04 (7.1%). We did not find any nasopharyngeal cancer in Vietnam, which resulted in the association between the HLA-DQB1 allele and patients with NPC. The results in this study were similar to those of other studies, such as that of Delfitri (2011) in Indonesia, which recorded high frequencies of the HLA-DQB1 alleles -DQB1*03, -DQB1*05, -DQB1*06, and -DQB1*02, whereas the -DQB1*04 allele was not found [24]. In addition, researchers (Wang and Wang, 2014) in northern China (Han ethnic group) recorded high frequencies for -DQB1*03 (59.1%), -DQB1*06 (37.9%), -DQB1*05 (33.3%), -DQB1*02 (25%), and -DQB1*04 (12.9%) [12].

Similarly, in the HLA-DQA1 locus, our study also detected five types of HLA-DQA1 alleles, and the most frequently found alleles were -DQA1*01 (35.7%), -DQA1*03 (28.6%), -DQA1*06 (21.4%), and -DQA1*02 (10.7%); the frequency of -DQA1*04 accounted for the lowest rate (3.6%). We did not find frequencies of HLA-DQA1 alleles in patients with NPC in either domestic or foreign studies. Therefore, it was impossible to compare and discuss this HLA gene locus.

In the pathogenesis of NPC, there are two main risk factors, namely 30 bp deletion mutations of the LMP1-EBV gene and the inherited factor of the HLA gene in NPC patients. The question continually posed in this study was whether these two risk factors in patients with NPC were associated. To answer this question, we continued to analyze the association between the 30 bp deletion mutation rate and the high frequencies of class-I HLA alleles (HLA-A and HLA- B) and class-II HLA alleles (HLA-DRB1, HLA-DQB1, and HLA-DAB1) in patients with NPC.

From Table 5, we could see no relationship between the 30 bp gene deletion mutation of LMP1-EBV and the high frequency of HLA-A alleles in the study sample (*p* > 0.05). Although the alleles HLA-A*02, HLA-A*11, HLA-A*24, and HLA-A*33 had high frequencies of occurrence in this study, these frequency results are similar to those of previous studies, both in Vietnam and abroad. According to the results of several previous studies, the HLA-A*02 allele plays an essential role in helping to present the LMP1-EBV antigen to CD8 T cells for the recognition and destruction of EBV-infected cells at the antigen-determining sites of YLLEMLWRL (125–133) and YLQQNWWTL (159–167) (Lin et al., 2004; Tang et al., 2008). A limitation in this study is that we only sequenced a short segment of the LMP1-EBV gene, the carboxyl terminus coding region, or CTAR2 (351–386), so it was not possible to make further judgments about this. However, the results of our study’s genetic-sequencing analysis showed that, in addition to the 30 bp gene deletion mutation of LMP1-EBV that the study results presented, the study also recorded the occurrence of many other nucleotide changes scattered throughout the LMP1-EBV gene (locations 168,225, 168,295, and 168,320). These results suggested that there might still be point mutations at other sites, particularly in the gene region encoding the CTAR1 segment of the LMP1-EBV molecule, which could help EBV escape the immune response of host translation and might be related to the HLA-A*02 allele. This HLA-A allele is responsible for presenting EBV antigens to recognized T cells. This suggests a new study direction for the pathogenesis of nasopharyngeal cancer related to the presence of HLA-A alleles and LMP1-EBV.

In addition, from Table 5, we compared the frequencies of HLA-B*15, HLA-B*38, and HLA-B*46 alleles, as well as high-proportion HLA-B alleles, in study samples with mutation rates of 30 bp LMP1-EBV gene deletion. Carriers of the HLA-B*15 allele (4.64; 1.23–17.52; 0.018) had a 4.6-times higher risk of losing 30 bp segment mutations of the LMP1-EBV gene than those who did not carry this allele. In particular, domestic and foreign studies have not published data on this association; this can be considered a new finding about the relationship between 30 bp gene deletion mutation of LMP1-EBV and the HLA-B*15 allele in NPC patients in Vietnam that is specific this study.

This study is an important first step in the process of researching LMP1-EBV gene expression in the biopsy tissue of patients with NPC using HLA-A, -B, -DRB1, -DQB1, and -DQA1 alleles investigated using a PCR-SSO technique. The novelty of this study is that it is the first study on the biology of nasopharyngeal cancer in the Mekong Delta, Vietnam. Our results provided scientific data on the rate of mutations in the LMP1-EBV gene for NPC tissue biopsy and the frequency of HLA alleles in Vietnamese patients, as well as on the relationship between the 30 bp gene deletion mutation of LMP1-EBV and HLA-B*15 alleles in NPC patients, which has not previously been studied, either in Vietnam or the world. This scientific document can be referred to in order to update teaching methods about nasopharyngeal cancer at universities of Medicine and Pharmacy. Moreover, these results can provide a premise for further studies on the biology of nasopharyngeal cancer in Vietnam, allowing clinicians to view the pathogenesis of NPC in patients more accurately, contributing to a more practical treatment effect.

## 5. Conclusions

Through 108 nasopharyngeal biopsy tissue samples of patients with NPC in southern Vietnam, we found that 72.9% of the LMP1-EBV genes had 30 bp deletion mutations. The HLA alleles appearing most frequently in patients with NPC were -A*02, 11, and 24; -B*15 and 46; -DRB1*12, 09, and 08; -DQB1*03, 05, and 06; and -DQA1*01, 03, and 06. Compared with those who did not carry the HLA-B*15 variant, carriers were more likely to experience 30 bp deletion on the LMP1-EBV gene. Our findings indicated that the majority of Vietnam’s NPC patients with EBV-associated carcinomas included the deleted version of LMP1. To further understand the LMP1 signaling system and determine the role that LMP1 sequence variation plays in the pathogenesis of EBV-associated malignancies, particularly NPC, more research using these variations is required.

## Figures and Tables

**Table 1:** Percentage of LMP1-EBV gene amplification products with a size of 200 bp detected in nasopharyngeal biopsy tissue samples of NPC patients studied by PCR technique and amplification product electrophoresis (*n* = 70).

LMP1-EBV Gene Amplification Products	Frequency (*n*)	Percentage (%)
Amplification product of 200 bp (30 bp deletion mutation)	51	72.9
Amplification product of 230 bp	19	27.1
Total	70	100

**Table 2:** Percentage of mutations with 30 bp deletion on LMP1-EBV gene by sequencing (*n* = 33).

LMP1-EBV Gene Mutation Type	Frequency (*n*)	Percentage (%)
30 bp deletion mutation (168,266–168,295)	25	75.8
No 30 bp deletion mutation (168,266–168,295)	8	24.2
Total	33	100

**Table 3:** Frequency of class-I HLA alleles (HLA-A and HLA-B) of studied patients with NPC.

No.	HLA-A Allele	Frequency (*n* = 52)	Percentage (%)	HLA-B Allele	Frequency (*n* = 52)	Percentage (%)
1	A*02	21	40.4	B*15	13	25.0
2	A*11	11	21.2	B*46	12	23.1
3	A*24	11	21.2	B*38	5	9.6
4	A*33	5	9.6	B*07	4	7.7
5	A*01	2	3.8	B*18	2	3.8
6	A*14	1	1.9	B*35	2	3.8
**7**	A*30	1	1.9	B*44	2	3.8
8				B*56	2	3.8
9				B*57	2	3.8
10				B*58	2	3.8
11				B*13	1	1.9
12				B*27	1	1.9
13				B*39	1	1.9
14				B*40	1	1.9
15				B*51	1	1.9
16				B*54	1	1.9

**Table 4:** Frequency of class-II HLA alleles (DRB1, DQB1, and DQA1) of studied patients with NPC.

No.	HLA-DRB1 Allele	Fre. (*n* = 58)	Per. (%)	HLA-DQB1 Allele	Fre. (*n* = 56)	Per. (%)	HLA-DQA1 Allele	Fre. (*n* = 56)	Per. (%)
1	DRB1*12	10	17.3	DQB1*03	25	44.7	DQA1*01	20	35.7
2	DRB1*09	8	13.8	DQB1*05	12	21.4	DQA1*03	16	28.6
3	DRB1*04	7	12.1	DQB1*06	10	17.9	DQA1*06	12	21.4
4	DRB1*08	7	12.1	DQB1*02	5	8.9	DQA1*02	6	10.7
5	DRB1*15	7	12.1	DQB1*04	4	7.1	DQA1*04	2	3.6
6	DRB1*14	6	10.3						
7	DRB1*07	6	10.3						
8	DRB1*10	3	5.2						
9	DRB1*03	1	1.7						
10	DRB1*06	1	1.7						
11	DRB1*13	1	1.7						
12	DRB1*16	1	1.7						

**Table 5:** Relationship between the rate of 30 bp deletion mutation of the LMP1-EBV gene and frequency of class-I HLA alleles in patients with NPC.

HLA	Mutation of 30 bp Deletion of LMP1-EBV Gene		OR	95% CI	*p*
	Mutation	Non-Mutation			
A*02 Non-A*02	**16 (47.1)** **18 (52.9)**	**5 (27.8)** **13 (72.2)**	0.43	0.13–1.48	0.178
A*11 Non-A*11	5 (14,7) 29 (85,3)	6 (33,3) 12 (66,7)	2,90	0.74–11.35	0.118
A*24 Non-A*24	7 (20.6) 27 (79.4)	4 (22.2) 14 (77.8)	1.10	0.28–4.42	0.891
B*15 Non-B*15	5 (14.7) 29 (85.3)	8 (44.4) 10 (55.6)	4.64	1.23–17.52	0.018
B*38 Non-B*38	4 (11.8) 30 (882)	1 (5.6) 17 (94.4)	0.44	0.05–4.27	0.729
B*46 Non-B*46	8 (23.5) 26 (76.5)	4 (22.2) 14 (77.8)	0.93	0.24–3.64	0.915
DRB1*12 Non-DRB1*12	7 (17.5) 33 (82.5)	3 (16.7) 15 (83.3)	0.94	0.21–4.16	0.938
DRB1*09 Non-DRB1*09	5 (12.5) 35 (87.5)	3 (16.7) 15 (83.3)	1.40	0.30–6.62	0.670
DQB1*03 Non-DQB1*03	11 (30.6) 25 (69.4)	5 (25.0) 15 (75.0)	0.76	0.22–2.61	0.659
DQA1*01 Non-DQA1*01	13 (36.1) 23 (63.9)	7 (35.0) 13 (650)	0.95	0.30–2.99	0.934

## Data Availability

Data sharing not applicable.
