# Correction: Trinh et al. LMP1-EBV Gene Deletion Mutations and HLA Genotypes of Nasopharyngeal Cancer Patients in Vietnam. *Pathophysiology* 2023, *30*, 1–12

**DOI:** 10.3390/pathophysiology31030025

**Published:** 2024-07-04

**Authors:** Cua Thi Hong Trinh, Dung Ngoc Tran, Linh Thi Thao Nguyen, Nghia Tin Tran, Minh Trinh Gia Nguyen, Vy Tran Phuong Nguyen, Nhung Thi Hong Vu, Khanh Duy Dang, Kha Van Vo, Hoa Chieu Chau, Phi Thi Phi Phan, Mai Huynh Truc Phuong

**Affiliations:** 1 Department of Pathophysiology and Immunology, Can Tho University of Medicine and Pharmacy, Can Tho 900000, Vietnam; 2 Department of Pharmacology and Clinical Pharmacy, Can Tho University of Medicine and Pharmacy, Can Tho 900000, Vietnam; 3 Can Tho Oncology Hospital, Can Tho 900000, Vietnam; 4 Can Tho Ear Nose Throat Hospital, Can Tho 900000, Vietnam; 5 Department of Physiopathology & Immunology, Ha Noi Medical University, Ha Noi 100000, Vietnam; 6 Faculty of Medicine, Can Tho University of Medicine and Pharmacy, Can Tho 900000, Vietnam

The authors would like to make the following corrections to the paper published [1] in *Pathophysiology* journal.

Regrettably, following publication, concerns were brought to the attention of the publisher regarding a possible overlap between the article [1] and two earlier articles [2,3] originating from the same authorship group and published in another language.

In accordance with our complaints procedure, an investigation was conducted by the editorial office and the institution of the corresponding author. Based on the extensive review by the institutional review board, academic editors, and reviewers, the decision was made to publish this correction.

In the original publication, there was a regrettable mistake in Figure 2 in the published paper [1], which also appeared in two papers published during the author’s doctoral studies [2,3]. The corrected Figure 2 appears below.

In the original publication [1], the two papers [2,3] should also have been cited and numbered as references [14,15] in Section 2.1 Materials, which now reads as follows:

“Nasopharyngeal biopsy tissue samples of NPC patients were obtained from Can Tho Oncology Hospital in the Mekong Delta region (from September 2014 to December 2018), as well as blood samples from NPC patients [14,15]”.

Subsequently, the references were re-numbered starting from the original reference [14], which should be reference [16] in the revised version.

Here, the three papers were briefly introduced, focusing on the differences. In the paper [2], 37 fresh samples were used to determine the ratio of expression of the LMP1 EBV gene by polymerase chain reaction assay. In the paper [3], 28 fresh and 37 new paraffin samples were collected to determine the frequency of the LMP1-EBV gene and the rate of a 30 bp loss mutant on the LMP1 gene (200 bp). A sequencing technique was applied to confirm LMP1 gene mutation in a few typical samples. In the *Pathophysiology* paper [1], the authors collected 37 fresh samples, 33 paraffin samples, 1 new fresh sample, and 37 new paraffin samples. A length of 30 bp of the del-LMP1-EBV gene was analyzed using a PCR technique. The HLA genotypes in patients’ blood samples were examined with PCR-SSO technology using a LABScan3DTM system (Luminex FLEXMAP 3D Version 4.2) with LABTypeTMXR and CWD Typing Test chemicals.

Additionally, after double-checking the authors’ contribution according to CrEdit taxonomy, we added Dr. Khanh Duy Dang’s and Dr. Mai Huynh Truc Phuong’s contribution to ‘assembly of data’ and ‘data analysis and interpretation’. The author contribution statement has been updated as follows:

“**Author Contributions:** Conception and design: C.T.H.T., L.T.T.N., N.T.T., M.T.G.N., V.T.P.N., and N.T.H.V.; provision of study material or patients: D.N.T., K.V.V., H.C.C., and P.T.P.P.; collection of data: C.T.H.T., D.N.T., L.T.T.N., N.T.T., and P.T.P.P.; assembly of data: C.T.H.T., D.N.T., L.T.T.N., N.T.T., P.T.P.P., K.D.D., and M.H.T.P.; data analysis and interpretation: C.T.H.T., L.T.T.N., N.T.T., M.T.G.N., V.T.P.N., K.D.D., M.H.T.P., and N.T.H.V.; writing—review and editing: C.T.H.T., L.T.T.N., N.T.T., M.T.G.N., V.T.P.N., N.T.H.V., D.N.T., P.T.P.P., K.V.V., H.C.C., K.D.D., and M.H.T.P. All authors have read and agreed to the published version of the manuscript.”

The authors state that the scientific conclusions are unaffected. This correction was approved by the editorial board and editor-in-chief. The original publication has also been updated.

## Figures and Tables

**Figure 2:**
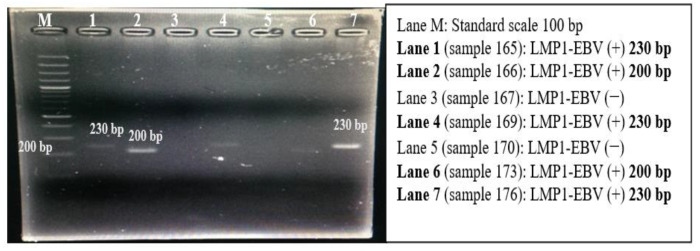
Electrophoresis line image of LMP1-EBV gene amplification product with a size of 200 bp (lane 2; lane 6) and 230 bp (lane 1; lane 4; lane 7) on 2% agarose gel.
